# Successful Management of Cervical Ectopic Pregnancy with Bilateral Uterine Artery Embolization and Methotrexate

**DOI:** 10.1155/2018/9593824

**Published:** 2018-05-03

**Authors:** Keitaroh Takeda, John Mackay, Susan Watts

**Affiliations:** Department of Emergency Medicine, Texas Tech University Health Sciences Center El Paso, 4801 Alberta Ave, Suite B3200, EL Paso, TX 79905, USA

## Abstract

Cervical ectopic pregnancy (CEP) is a rare form of ectopic pregnancy. Cases diagnosed early in pregnancy can be managed medically, but more advanced pregnancies often require hysterectomy. Uterine artery embolization (UAE) is a novel approach to CEP for those who wish to preserve fertility. Here we present the case of a 44-year-old female with a 2-week history of vaginal bleeding and abdominal pain who was diagnosed with CEP and successfully treated with bilateral UAE (BUAE) in combination with methotrexate. A 44-year-old female presented to the emergency department with a 2-week history of vaginal bleeding. Serum beta-hCG was 71,964 mIU/ml. The transvaginal ultrasound confirmed CEP. The patient was referred to obstetrics and interventional radiology and ultimately treated with BUAE and methotrexate. Symptoms resolved quickly and she was discharged after 3 days.

## 1. Introduction

Ectopic pregnancy is a life-threatening condition that must be ruled-out in any female presenting with vaginal bleeding and lower abdominal pain. Cervical ectopic pregnancy (CEP) is a rare form of ectopic pregnancy in which implantation occurs in the endocervical canal rather than in the uterus. Several treatment options have been described in the literature, including medical management with methotrexate (MTx) with or without KCL, but hysterectomy is indicated if medical management fails or if the woman is hemodynamically unstable [1]. Uterine artery embolization (UAE) is a novel treatment approach for those who wish to preserve fertility. Here we report a case of cervical ectopic pregnancy successfully managed with UAE and methotrexate.

## 2. Case Presentation

**Figure 1:**
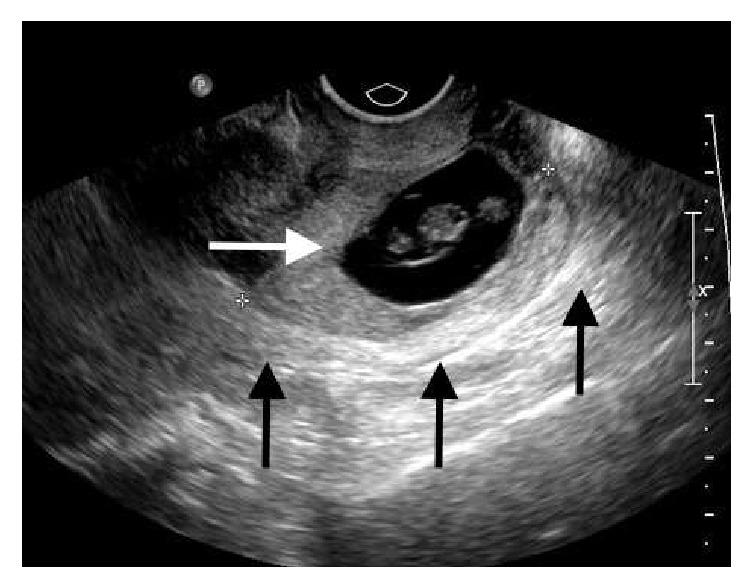
Transvaginal ultrasound showing a live ectopic pregnancy (white arrow) in the posterior wall of the cervix (black arrows). FHR 174 pbm. Estimated fetal age was 8 weeks, 1 day.

A 44-year-old G2P1011 female with no significant past medical history presented to the emergency department (ED) with a 2-week history of vaginal bleeding. The patient also complained of mild fatigue and dizziness along with left lower quadrant abdominal pain. Her last menstrual period was 8 weeks prior to the ED visit. The patient was tachycardic (heart rate 138 beats per minute) with otherwise normal vital signs. On physical examination, the patient had mild suprapubic tenderness, and the pelvic examination showed a closed external os with active vaginal bleeding. Her hemoglobin and hematocrit were 10.0 g/dL and 29.5%, respectively; serum beta-hCG was 71,964 mIU/mL. The preliminary transvaginal ultrasound report indicated an 8-week intrauterine pregnancy with cardiac activity, but the subsequent official report by a radiologist confirmed a cervical ectopic pregnancy (CEP) located at the posterior wall of the cervical canal (Figure 1). The patient's tachycardia did not improve despite 2 L normal saline bolus, so she was given 2 units of packed red blood cells (PRBCs).

**Figure 2:**
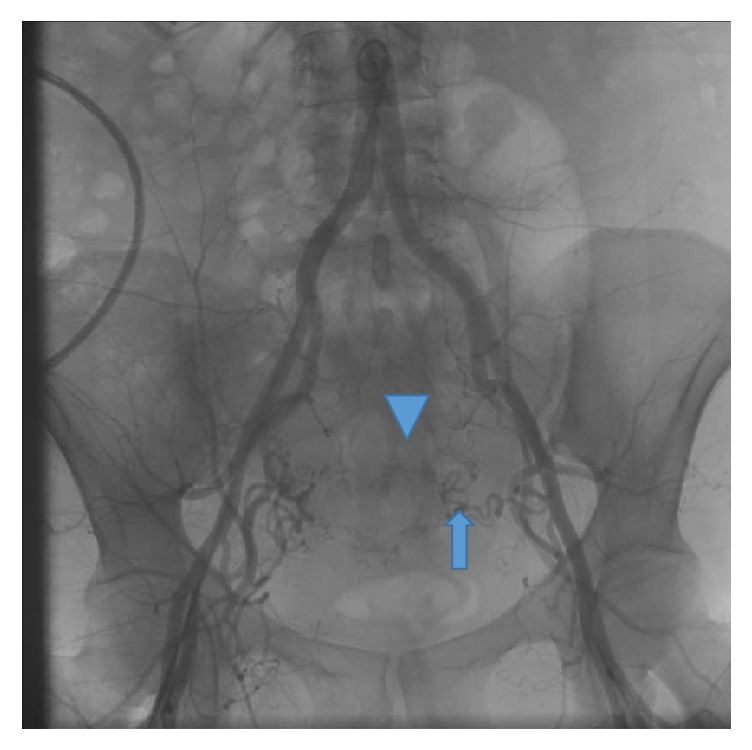
Pelvic aortogram showing a hypervascular area in the region of the cervical ectopic pregnancy (arrowhead) being supplied mainly by the left uterine artery (arrow).

**Figure 3:**
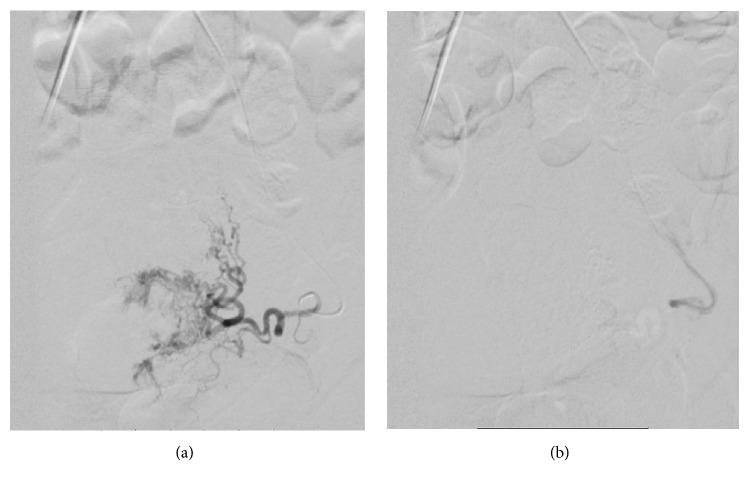
Pre- and postembolization arteriogram showing the bilateral uterine arteries (including the cervical component) prior to embolization with Gelfoam and after ((a) preembolization and (b) postembolization).

Due to the hypervascularity of the cervical ectopic pregnancy, the obstetrics consultant recommended uterine artery embolization (UAE) via interventional radiology. The pelvic angiogram revealed that the ectopic pregnancy was supplied mainly by the left uterine artery (Figure 2); however the ascending components of both uterine arteries were selected with a microcatheter and embolized with Gelfoam (9 cc for right and 6 cc for left, resp.). Postembolization images showed satisfactory occlusion of blood flow (Figure 3).

The patient was stable after BUAE and was transferred to the floor. In addition, intramuscular methotrexate (MTx) 1 mg/kg was given on day 1. Consequently, vaginal bleeding stopped, beta-HCG dropped to 16,086 mIU/mL, and her vital signs remained within normal limits. She was given folic acid on day 2 to neutralize the effects of methotrexate. Her remaining hospital course was uncomplicated and her beta-HCG trended down to 3,646 mIU/mL on day 3. She received a second dose of methotrexate 1 mg/kg IM on day 3 and was discharged home with no further vaginal bleeding. On follow-up day 35, the patient's beta-HCG was less than 1 mIU/ml. All symptoms had resolved and no further intervention was required. The patient was last seen in the clinic on day 51 for IUD placement. Her menstrual cycle was not reported.

## 3. Discussion

CEP is the second rarest form of ectopic pregnancy following abdominal ectopic pregnancy [2] with a reported incidence of 1 in 1,000–18,000 pregnancies [3]. The etiology of CEP is not fully understood but reported risk factors for CEP include history of pelvic inflammatory disease, smoking, previous pelvic surgery, previous ectopic pregnancy, intrauterine device use, anatomic anomalies, previous cesarean delivery, previous uterine or cervical surgery, in vitro fertilization, and diethylstilbestrol exposure [2]. In an article by Paalman, 5 clinical signs of cervical ectopic pregnancy were identified: (1) uterine bleeding without cramping pain after a period of amenorrhea, (2) softened and disproportionately enlarged cervix equal to or larger than the corporal portion of the uterus (an hourglass-shaped uterus), (3) products of conception entirely confined within, and firmly attached to, the endocervix, (4) a snug internal os, and (5) a partially opened external os [2]. The diagnosis of CEP is established by transabdominal and/or transvaginal ultrasound. Sonographic diagnostic criteria are (1) empty uterine cavity or thickened endometrium, (2) distended and/or enlarged cervix, (3) gestational sac or placental tissue below the level of the internal os, (4) negative “sliding organs sign”, and (5) high peritrophoblastic vascularity on Doppler examination (peak velocity > 20 cm/s, pulsatility index < 1.0) [4].

CEP is traditionally considered as high risk for hemorrhage and has historically been treated with hysterectomy, leading to loss of fertility [5]. With improvements in ultrasound, early diagnosis of CEP is possible, allowing for the opportunity to use conservative medical management and interventional measures rather than surgical management [3]. Medical management options include systemic or local injection of methotrexate, KCL, local vasopressin injection, local or systemic prostaglandin, systemic mifepristone, and intrauterine irrigation with 3.5% H_2_O_2_ [2]. The factors that favor conservative medical management are early diagnosis preferably before 12 weeks, low beta-hCG levels, and absence of cardiac activity [2, 6]. However, hemodynamically unstable patients or those who fail medical management require hysterectomy. Verma has reported a case series of 24 CEP cases treated successfully with medical management alone. In this case series, no patient required hysterectomy [7]. Traditionally, UAE has been used to control bleeding in pelvic trauma and bleeding from cervical cancer or as a treatment for uterine fibroid. UAE for CEP was initially reported as an adjuvant measure to decrease blood loss associated with dilation and curettage (D&C) [8]. UAE has reportedly been used in combination with medical management, curettage, and office hysteroscopic resection for treatment of CEP [2, 7, 9, 10]. To date, there are no established criteria for the use of UAE in CEP. In an article by Zakaria et al., the indication for UAE is as follows: (1) initial beta-hCG is > 34,000 mIU/mL; (2) patient is unable to tolerate methotrexate and leucovorin (MTx/Leu) [10]. Our patient had no risk factors for CEP. Although the preliminary ultrasound report suggested intrauterine pregnancy, the diagnosis of CEP was confirmed by a radiologist. The fact that patient was hemodynamically stable after 2 U of PRBCs favored conservative medical management, rather than hysterectomy. According to the criteria proposed by Zakaria et al., the patient's beta-hCG level over 34,000 mIU/mL made her a candidate for MTx plus UAE treatment [10].

The benefit of UAE in the treatment of CEP is preservation of fertility. However, there are adverse effects associated with UAE including uterine infarction, ischemia or necrosis, sciatic nerve injury, and necrosis of the bladder or the rectum [8, 10]. In one case series, 1 out of 3 patients required hysterectomy due to necrosis of the uterine myoma after BUAE [5]. Patient education should include information about potential loss of fertility after UAE. Further studies are needed to evaluate the effect of UAE on fertility [11].

## 4. Conclusion

This case describes a case of cervical ectopic pregnancy treated with conservative management using systemic methotrexate in combination with BUAE. This case illustrates 2 important issues regarding management of CEP.

First, CEP is sometimes misdiagnosed as intrauterine pregnancy. In this case, the first preliminary report suggested intrauterine pregnancy. Although CEP is very rare, it poses a high risk for hemorrhage. CEP should be excluded in all suspected intrauterine pregnancies.

Second, UAE can be used as part of urgent minimally invasive treatment of cervical ectopic pregnancy. This patient was managed with BUAE in combination with systemic methotrexate. The vaginal bleeding subsided and beta-hCG steadily declined with the treatment. Our patient was discharged home in 3 days without complications. If interventional radiology facilities are available, emergency physicians should consider this novel approach to CEP in consultation with obstetricians and interventional radiologists.
